# Alternative Access for TAVR: Choosing the Right Pathway

**DOI:** 10.3390/jcm13123386

**Published:** 2024-06-09

**Authors:** Katherine Lutz, Karla M. Asturias, Jasmine Garg, Abhushan Poudyal, Gurion Lantz, Harsh Golwala, Julie Doberne, Amani Politano, Howard K. Song, Firas Zahr

**Affiliations:** 1 Division of Cardiology, Knight Cardiovascular Institute, Oregon Health & Science University, Portland, OR 97201, USA; lutzka@ohsu.edu (K.L.); asturiak@ohsu.edu (K.M.A.); poudyal@ohsu.edu (A.P.); golwala@ohsu.edu (H.G.); 2 Department of Medicine, Westchester Medical Center, Valhalla, NY 10595, USA; jasminegarg1995@gmail.com; 3 Division of Cadiothoracic Surgery, Knight Cardiovascular Institute, Oregon Health & Science University, Portland, OR 97201, USA; lantz@ohsu.edu (G.L.); oberne@ohsu.edu (J.D.); songh@ohsu.edu (H.K.S.); 4 Division of Vascular Surgery, Knight Cardiovascular Institute, Oregon Health & Science University, Portland, OR 97201, USA; politano@ohsu.edu

**Keywords:** transcatheter aortic valve replacement, alternative access, vascular access, transapical, transaortic, transaxillary, transsubclavian, transcarotid

## Abstract

Transcatheter aortic valve replacement (TAVR) has emerged as an alternative treatment option for patients with severe aortic stenosis regardless of surgical risk, particularly in those with a high and prohibitive risk. Since the advent of TAVR, transfemoral access has been the standard of care. However, given comorbidities and anatomical limitations, a proportion of patients are not good candidates for a transfemoral approach. Alternative access, including transapical, transaortic, transaxillary, transsubclavian, transcarotid, and transcaval, can be considered. Each alternative access has advantages and disadvantages, so the vascular route should be tailored to the patient’s characteristics. However, there is no standardized algorithm when choosing the optimal alternative vascular access. In this review, we analyzed the evolution and current evidence for the most common alternative access for TAVR and proposed an algorithm for choosing the optimal vascular access in this patient population.

## 1. Introduction

**Figure 1:**
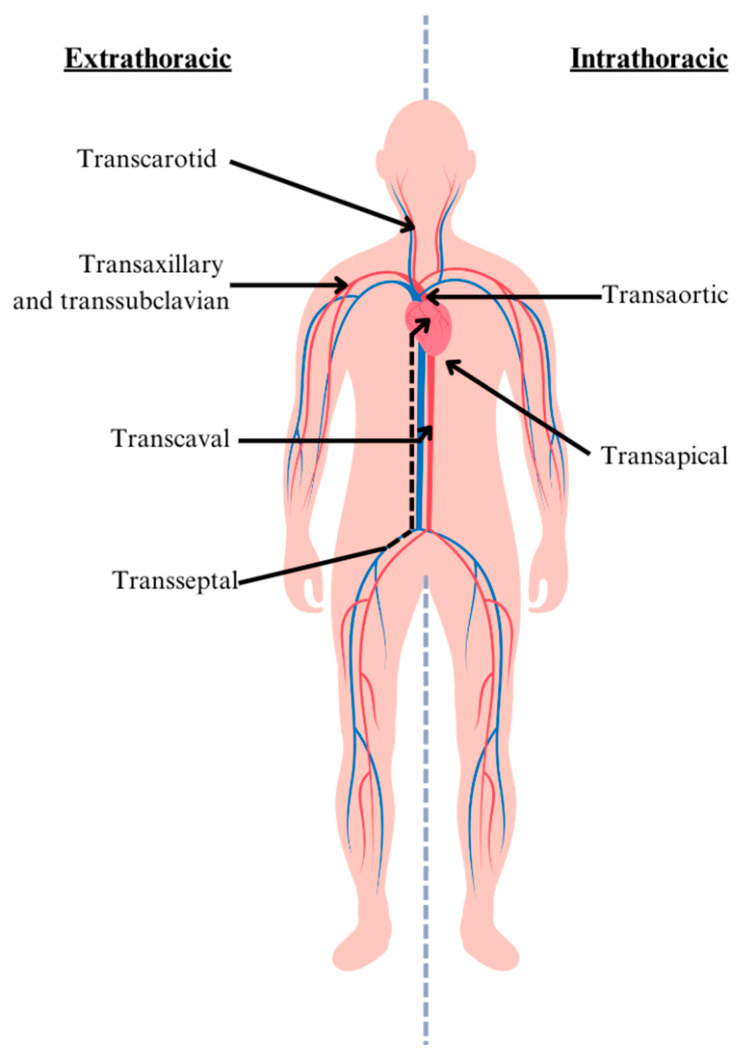
Alternative TAVR accesses. Most common intra- and extrathoracic alternative access approaches for TAVR.

Transcatheter aortic valve replacement (TAVR) has revolutionized the management of severe aortic stenosis, offering an alternative to surgical aortic valve replacement in a variety of patients. Previously, TAVR was recommended for those with high or prohibitive surgical risk; however, TAVR can now be offered as a standard of care regardless of surgical risk [1,2]. The femoral artery is the most widely adopted vascular access route for TAVR; however, despite reduced sheath sizes and improved device technology over the years, up to 5–12% of patients require alternative access [3,4]. This is mostly due to severe peripheral artery disease (PAD), tortuous iliofemoral arteries, calcified lesions, aortic aneurysm, or previous vascular surgeries. In these cases, alternative accesses are preferred, with the most common accesses illustrated in Figure 1.

In a retrospective analysis from the Society of Thoracic Surgeons/Transcatheter Valve Therapy Registry, patients with PAD who were undergoing transfemoral TAVR had a higher incidence of death, readmission and bleeding when compared with patients without PAD. Notably, this trend was not observed in patients who underwent TAVR via alternative access [5]. This underscores the need for TAVR centers to adopt a streamlined and multidisciplinary approach to alternative access for TAVR. In this review, we will describe the evolution of these techniques, advantages, disadvantages, and suggest an algorithm when selecting vascular access site.

### 1.1. Evolution of Alternative Accesses for TAVR

**Figure 2:**
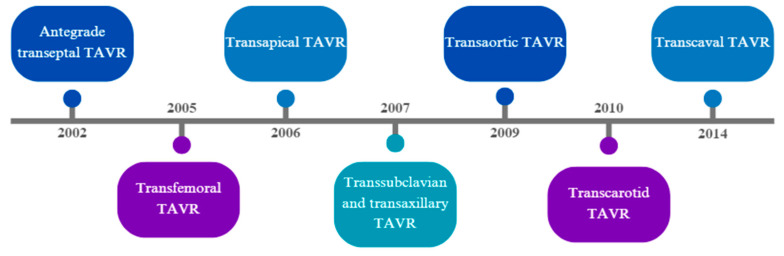
Evolution of TAVR access. Timeline showing year of first report for each TAVR access.

TAVR has undergone a significant evolution over the past two decades, highlighted in Figure 2. The first TAVR procedure in humans was reported in 2002, using an antegrade transseptal approach in a patient with severe aortic stenosis, multiple comorbidities, and cardiogenic shock, who had a prior balloon aortic valvuloplasty with restenosis [6]. The first retrograde TAVR in humans using a transfemoral arterial access was performed in Venezuela in 2005 in a patient with severe aortic stenosis and acute decompensated heart failure refractory to medical therapy [7]. Shortly after, in 2006, the first case of transapical TAVR was performed in a patient with severe aortic stenosis, with prohibitive surgical risk, that was not a candidate for transfemoral access due to a large infrarenal aortic aneurysm and bilateral aortoiliac disease [8].

Two registries published in 2004 and 2006 described the early outcomes of TAVR, with most procedures performed via an antegrade transseptal approach [9,10]. Despite this procedure offering a novel therapy for patients who were at high surgical risk for TAVR, the transseptal approach carried high procedural complexity, a need for atrial septal crossing and a risk of injury to the anterior mitral valve leaflet with subsequent mitral regurgitation. This approach is still occasionally used as a bail-out strategy for alternative access when all other approaches have been explored, particularly as newer technology for atrial septal defect closure devices has been developed, but has largely been replaced by other alternative options [11,12]. However, due to the risks involved in antegrade septal crossing, attention was mainly focused on the refinement of other approaches, particularly the transfemoral approach [13].

As TAVR became more common, the need for additional alternative accesses apart from the transfemoral approach became evident due to challenges with severe PAD due to atherosclerosis and calcifications, tortuosity of the iliofemoral system, and the presence of thoracic or abdominal aortic aneurysms. The first of these approaches were the transsubclavian and transaxillary, published for the first time in 2007 [14,15]. The transaortic and transcarotid approaches were first described in 2009 and 2010, respectively, both in patients with severe iliofemoral and subclavian PAD [16,17]. A few years later, in 2014, transcaval access was described in patients who were believed not to have other access options [18]. The procedural aspects, advantages, disadvantages, and outcomes of each alternative access will be discussed in this review.

The frequency of use of alternative accesses in TAVR has changed over the past decade, with an overall trend of decreased use of transapical access and increased utilization of transcarotid, transaxillary, and transsubclavian accesses. An analysis from the FRANCE 2 registry that included 4165 patients enrolled between January of 2010 and January of 2012 reported a transapical access rate of 17.8% and subclavian access rate of 5.8%. The FRANCE TAVI registry that included 12,804 patients enrolled from January of 2013 to December of 2015 reported significantly lower transapical access use, 4.2%, and subclavian access, 3.0%, with an increase in other types of access, including transaortic, transaxillary, and transcarotid [19]. In a report from the Society of Thoracic Surgeons/American College of Cardiology Transcatheter Valve Therapy Registry that included 7187 patients who underwent alternative access TAVR between June 2015 and June 2018, 52% had peripheral access and 48% had central access. Of the peripheral access patients, 87.6% had transaxillary/transsubclavian access and 12.4% underwent transcarotid access. For the central access patients, the transpical and transaortic frequencies were 56.1% and 43.9%, respectively [20].

### 1.2. Transfemoral Access and Access Site Modification

The transfemoral approach remains the gold standard approach for vascular access in TAVR. Early on, surgical cutdown was the most common method for accessing the femoral artery, but there has been a shift to percutaneous access over time [21]. This transition happened most likely due to substantially decreased sheath sizes from 22–24 Fr to 14 Fr, most commonly used nowadays, minimizing the need for routine vascular expertise and general anesthesia, as are often needed for surgical cutdowns. Additionally, increased experience with vascular closure devices for larger arteriotomy sites and the incentive to reduce the length of stay promote use of a less invasive percutaneous strategy [22]. Several studies have compared transfemoral surgical and percutaneous access. One study found similar major and minor complications, while another found higher rates of minor vascular complications and lower rates of bleeding in the percutaneous access group [23,24]. One retrospective study showed that percutaneous access was associated with higher rates of isolated stenosis or dissection, while surgical cutdown was associated with higher rates of wound infections and blood transfusions [25]. In order to avoid vascular complications, surgical cutdown may still be preferred in certain patient populations including those with a large body habitus, prior aortofemoral grafts, and large amounts of calcium that would result in the failure of a vascular closure device.

Adjunctive therapies for the optimization of femoral access with access site modification have also been utilized. Predilation of small caliber vessels in patients with PAD has been performed with ballon angioplasty and a balloon-expandable sheath to facilitate transfemoral access [26]. Lithotripsy has been utilized for calcium modification of PAD and has been shown to be safe and effective when used to enable large bore sheath advancement through calcified iliac arteries in the Disrupt PAD III study [27]. Intravascular lithotripsy has also been studied in observational studies and in a prospective registry and has been shown to be safe and effective, although the patients had a higher than average incidence of periprocedural complications [28,29]. Orbital atherectomy has been successfully utilized as an adjunct for optimizing transfemoral access in the absence of alternative access options [30].

## 2. Intrathoracic Alternative Accesses

### 2.1. Transapical Access

#### 2.1.1. Procedural Technique

Transapical access usually requires general anesthesia, as it involves an anterolateral left mini thoracotomy that allows exposure of the left ventricular apex after opening the pericardium. Two concentric purse-string sutures are placed, and anticoagulation is administered. A transapical sheath is placed over the wire through the apex into the left ventricle, and the valve is deployed under rapid ventricular pacing with image guidance. Rapid ventricular pacing is also used during the sheath removal and suture tying to reduce pressure until the repair is completed. Of note, this access cannot be used to deploy self-expanding valves as they are premounted on the delivery system in a way that prevents antegrade delivery [31,32].

#### 2.1.2. Outcomes

Observational retrospective and prospective studies have associated transapical TAVR with worse outcomes when compared to transfemoral TAVR. However, an argument has been made that transapical TAVR groups tend to have more comorbidities and risk factors than patients undergoing transfemoral accesses [33]. A meta-analysis that included 2978 patients with severe aortic stenosis and high surgical risk compared transfemoral TAVR (1465 patients) with transapical TAVR (1513 patients), showing increased 30-day all-cause mortality and major vascular events in the transapical group when compared to the transfemoral group, but with a similar 1-year mortality [34].

A study from the PARTNER I trial that included 1100 patients that underwent transapical TAVR and 1521 patients that underwent transfemoral TAVR attempted to neutralize patient differences using propensity score matching. Matched transapical TAVR patients experienced more adverse procedural events, a longer length of stay, a higher incidence of blood transfusions, a slower recovery, a higher operative, in-hospital, and 6-month mortality, and a lower incidence of aortic regurgitation when compared to transfemoral patients [35].

The PARTNER II trial randomized 2032 patients with symptomatic severe aortic stenosis at intermediate perioperative risk to TAVR or surgical AVR (SAVR) with the primary composite endpoint of death from any cause and disabling stroke at 2 years. Along with a comparison of TAVR and SAVR, it provided insight into transfemoral and transthoracic TAVR outcomes. In the transfemoral access cohort, TAVR resulted in a lower incidence of the primary endpoint when compared to SAVR. On the other hand, the transthoracic access cohort (comprised of 174 transapical and 62 transaortic accesses) showed no significant difference compared to SAVR [36].

The STACCATO trial, a prospective, randomized controlled trial of transapical TAVR versus surgical AVR in low-risk operative patients with aortic stenosis was terminated early after the inclusion of 70 patients due to an excess of events in the transapical group after the primary endpoint was met in 5 patients (two deaths, two strokes, and one case of renal failure requiring hemodialysis) [37]. Sohal et al. reviewed the National Inpatient Sample database from 2011 to 2017 and identified a down-trend in the utilization of the transapical approach for TAVR from a peak of 27.7% in 2013 to 1.92% in 2017. This finding coincided with a peak in inpatient mortality from transapical access TAVR in 2014 at 5.53% which decreased to 3.18% in 2017, while the inpatient mortality for non-transapical access TAVR was 3.02% in 2014 and 1.24% in 2017 [38].

### 2.2. Patient Selection

Of the alternative accesses, the transapical is considered one of the most invasive approaches as a thoracotomy, a puncture of the left ventricular apex, general anesthesia, and mechanical ventilation are required. The procedure comes with the inherent risks of crossing the left ventricular apex with the delivery system, which may be associated with myocardial stunning, possible necrosis or formation of a left ventricular aneurysm, impacting the left ventricular function. Other risks include damage to the mitral valve chordae that could result in acute mitral insufficiency [33]. Additionally, in patients with chronic obstructive pulmonary disease, longer postprocedural ventilation times have been noted in transapical TAVRs when compared to transfemoral approaches [39]. For these reasons, patients should be considered for this approach only if all extrathoracic options have been exhausted and there is significant calcification of the ascending aorta, precluding transaortic access.

### 2.3. Transaortic Access

#### 2.3.1. Procedural Technique

Transaortic access involves a partial sternotomy incision or mini thoracotomy performed through the second, third, or fourth intercostal space, according to the position of the aorta on preprocedural imaging. This is followed by pledgeted purse-string suture placement in the distal ascending aorta, obtaining needle access, sequential dilation, sheath delivery, and valve deployment under rapid ventricular pacing with image guidance. When finished, the purse strings are tied under a direct visualization [32]. This procedure is generally performed under general anesthesia and does not have a percutaneous option.

#### 2.3.2. Outcomes

Observational data with high-risk patients suggest that transaortic TAVR has lower short- and long-term mortality when compared to the transapical approach [33]. One small study in France compared outcomes among 467 cases of transfemoral, 42 cases of transapical, and 289 cases of transaortic approaches between 2011 and 2014. Although not significant, the cumulative 1-year survival rate tended to be higher in the transfemoral group compared to the transaortic group. Similarly, although not statistically significant, the cumulative 1-year survival rate for the transaortic approach was higher compared to the transapical approach [40]. In a propensity score matched retrospective study of 394 matched pairs of transfemoral access and transaortic access, all-cause mortality, stroke, major bleeding, and acute kidney injury were significantly higher at 30 days in the transaortic group [41]. A meta-analysis consisting of case series compared patients with severe aortic stenosis undergoing transapical and transaortic TAVR. Thirty-day mortality was similar at 7.9% for transaortic and 9.7% for transapical approaches. There was a non-significant trend toward a lower rate of stroke in the transaortic when compared to the transapical approach (0.9% vs. 2.1%, respectively), with similar rates of other complications [42].

These data suggest a possible superiority of transaortic access compared to transapical access. However, transfemoral and extra-thoracic alternative access sites are preferable to transaortic access. Compared to the transfemoral approach, the transaortic approach results in higher complication rates, including major life-threatening bleeding at 30 days as high as 66.7% after transaortic access [41]. A network meta-analysis that included 34 studies with a pooled sample size of 32,689 patients found that both transaortic and transapical access were associated with a 2-fold increase in 30-day mortality and a 1.4-fold increase in 1-year mortality when compared to transfemoral access. Extra-thoracic sites (transaxillary/transcubclavian, transcarotid, and transcaval) demonstrated comparable mortality and stroke risk to transfemoral access in this network meta-analysis [43].

#### 2.3.3. Patient Selection

Transaortic TAVR should be utilized in patients in whom transfemoral access is not possible and extrathoracic alternatives have been deemed unsuitable for the patient. Features that preclude the use of transaortic access include thorax deformities, a very short ascending aorta, porcelain aorta, and the presence of patent venous coronary artery bypass graft with proximal anastomosis on the ascending aorta. Careful inspection of the pre-TAVR CT scan to ensure 1 square cm is free of calcium where the purse string sutures will be placed is crucial. The trajectory of the aorta must be evaluated, and more horizontal aortas with an angle greater than 70% risk valve malalignment. Aortic entry should be at least 6 cm above the aortic annulus to ensure enough room for the delivery system [44].

## 3. Extrathoracic Alternative Accesses

### 3.1. Transaxillary and Transsubclavian Access

#### 3.1.1. Procedural Technique

Transaxillary access can be performed through surgical exposure for the sheath insertion or as a complete percutaneous access. Typically, general anesthesia is utilized for patient comfort if surgical cutdown is pursued, while conscious sedation could be considered in percutaneous access. The left axillary artery is typically preferred, as it provides a more direct pathway and optimal valve coaxial alignment within the aortic annulus. Contralateral radial access is performed to facilitate the introduction of a cerebral protection device. Femoral arterial and venous access are also obtained to perform right ventricular pacing and aortic root ventriculography, respectively. In a surgical technique, the artery is exposed through an infraclavicular incision, purse-string sutures are placed, the artery is punctured in the center of the purse strings, heparin is given, wire access is obtained, and the sheath is placed. Operators should aim to access the distal end of the first segment or proximal second segment, near the medial border of the pectoralis minor muscle and just proximal to the thoracoacromial branch. This site is optimal due to the invariant position of the artery, absence of overlying nerves and veins, and the potential ability to compress the artery against the second rib. In a percutaneous approach, the second segment is preferred to avoid pneumothorax and potentially use manual pressure against the rib. Similar to surgical access, the second segment of the axillary artery is the preferred location for percutaneous puncture. In addition to the previously mentioned reasons, accessing the second segment reduces the risk of pneumothorax and allows for proximal control if open surgical repair becomes necessary. Moreover, the placement of covered stents and the deployment of closure devices are safer at this location. Percutaneous access can be facilitated by digital subtraction angiography, road mapping, or ultrasound guidance. If fluoroscopy is being used for access, a wire can be placed into the subclavian artery from the femoral access to guide the access site [45]. Following axillary artery puncture, the artery is dilated, and Proglide sutures are implanted before large-sheath insertion. A more detailed description of the technique is beyond the scope of this paper.

After either percutaneous or surgical transaxillary access is achieved, the aortic valve is crossed, and the valve is deployed under rapid ventricular pacing with image guidance. Hemostasis is achieved by primary repair (in the surgical technique) or tightening of Perclose sutures and a short-duration balloon tamponade (in the percutaneous technique) [32,46].

#### 3.1.2. Outcomes

Multiple retrospective studies have compared the transaxillary with the transfemoral access, showing similar procedural outcomes, but most data include self-expanding valves and a surgical cutdown technique. Among patients enrolled in the Italian CoreValve Registry between June 2007 and March 2011, a prospective propensity-matched analysis comparing the femoral approach and the subclavian approach described similar procedure success, major vascular complications, life-threatening bleeding, and survival at 2 years [47]. In a propensity-weighted comparison from the Cleveland Clinic to evaluate outcomes of alternative access approaches, transaxillary TAVR was compared to intrathoracic approaches (transapical and transaortic) and was associated with fewer blood product transfusions, a shorter mechanical ventilation time, less postoperative atrial fibrillation, a shorter hospital stay, a higher likelihood of extubation in the operating room, and similar incidence of stroke [32]. Similar outcomes were described from the Ochsner Medical Center, which performed a retrospective propensity-adjusted analysis of patients undergoing transapical or transaxillary TAVR. The shorter time to extubation, lower reintubation rates, and lower in-hospital length of stay were statistically significant, favoring the transaxillary TAVR group [48]. Based on the studies available, transaxillary TAVR access seems to be a safe and feasible alternative access site in carefully selected patient populations.

#### 3.1.3. Patient Selection

Pre-procedure CT scan is crucial for determining candidacy for transaxillary alternative access. Factors to consider for patient selection for the transaxillary approach include the caliber of axillary and subclavian arteries (preferably greater than 6.5 mm), the presence of calcifications, tortuosity, ipsilateral dialysis fistula, and the location of side branches along the artery [44]. In addition, large-bore axillary access should be avoided in the presence of an ipsilateral patent internal mammary graft, as its dissection or occlusion during decannulation can be associated with serious complications. When compared with the femoral arteries, the subclavian and axillary arteries tend to have more layers of elastic fibers in the media, instead of more smooth muscle, making subclavian and axillary arteries more prone to dissection [49]. Occasionally, the presence of an ipsilateral pacemaker or defibrillator encroached in the deltopectoral groove could make access more challenging [46].

### 3.2. Transcarotid Access

#### 3.2.1. Procedural Technique

Transcarotid access is usually performed in a hybrid operating room. Most cases are performed through surgical cutdown, but there have been two reported cases of successful percutaneous transcarotid TAVR access [49]. General anesthesia is most commonly used, but conscious sedation with local anesthesia has been utilized successfully [50,51]. For the surgical cutdown approach, the common carotid artery is surgically exposed, with silicone vessel loops placed proximally and distally around the vessel. Systolic blood pressure is maintained at no lower than 120 mmHg, administering pressors when necessary. Next, the proximal common carotid artery is clamped, a small needle is introduced distally to the clamp, and the mean arterial pressure (MAP) is recorded. A distal perfusion MAP of greater than 32 mmHg is required. If this pressure is not obtained despite chemical hemodynamic maneuvers, a 5-min test clamp is performed with cerebral saturation monitoring, and the procedure is aborted if the cerebral saturation declines by more than 20%. Next, a micropuncture needle is used to enter the carotid artery, and a wire is threaded down the aorta and exchanged for a small sheath. In contrast to other approaches, the valve is crossed with a smaller catheter before the large sheath is introduced to minimize carotid occlusion from the sheath. Loops are then tightened above and below the sheath, or the distal common carotid artery is clamped to prevent distal embolization during valve delivery. The smaller sheath is then removed, a transverse arteriotomy is performed, the valve delivery sheath is introduced over the wire without dilation, and the valve is deployed as per protocol. Finally, the carotid artery is primarily closed [52].

#### 3.2.2. Outcomes

There has been concern with transcarotid access site and stroke incidence compared to transfemoral and transaxillary approaches. A multicenter study conducted in Canada and France from 2012 to 2017 used propensity score analysis to compare 101 patients with transcarotid TAVR access with 228 patients with invasive chest TAVR access (transapical and transaortic access), and demonstrated that transcarotid access was associated with a decreased new onset of atrial fibrillation, major bleeding, acute kidney injury, and length of stay. Similar rates of stroke and 30-day all-cause mortality occurred between the invasive chest access and transcarotid access groups [53]. A single high-volume center in Oregon conducted a propensity score weighted study, comparing 146 cases of transcarotid to 1465 cases of transfemroal TAVR access and demonstrated a similar procedure time, length of stay, 30-day stroke, and mortality, as well as major vascular complications and survival rates [54].

A single-center prospective study compared transapical, transfemoral, and transcarotid access outcomes. Although the sample size only contained 25 patients in the transcarotid group, the results suggest that transcarotid and transfemoral access had lower procedural times and shorter intensive care unit and hospital length of stays when compared to transapical access. No transcarotid access patients had in-hospital stroke or TIA, and only one had TIA at the 30-day follow-up [52]. Another prospective multicenter French study evaluating outcomes of the transcarotid access enrolled 314 patients across 13 centers, and ultimately had a stroke rate of at 1.6% at 30 days, which is lower than observed in the PARTNER 2 trial at 5.5% consisting primarily of transfemoral access [55].

#### 3.2.3. Patient Selection

Transcarotid access represents a direct pathway to the aortic valve, offering a shorter distance from the entry site to the aortic root when compared to other access strategies. The shorter route translates into better device control and positioning during the valve implantation. Despite the initial major concerns of this approach and the potential risk of stroke due to embolic events from carotid atherosclerotic plaques and calcifications, outcomes have not shown higher rates of strokes when compared to the transfemoral approach. However, patient selection and thorough imaging review are essential and heavy tortuosity, atheromas, and calcifications should lead to consideration of other TAVR approaches.

Preprocedural planning often requires carotid and vertebral Doppler ultrasound and CTA or MRI of the brain to confirm patency of the circle of Willis and collateral cerebral blood flow, in addition to the usual pre-TAVR imaging. In addition to assessment for carotid tortuosity and calcifications, the size of the carotid artery and presence of significant contralateral carotid stenosis should be evaluated, as a contralateral stenosis > 50% is considered a contraindication. The ideal carotid diameter should be preferably greater than 6.5 mm, and should accommodate the planned sheath size. Additional potential contraindications to transcarotid access include prior ipsilateral carotid artery intervention and stenosis or occlusion of the vertebral arteries [56].

### 3.3. Transcaval Access

#### 3.3.1. Procedural Technique

Conscious sedation is typically utilized for the procedure. The femoral vein and the largest femoral artery are both accessed percutaneously using a modified Seldinger technique. After vascular access is obtained, anticoagulation is given, and an aortogram is performed. A coaxial crossing system is used, and a snare is positioned in the aorta with confirmation in orthogonal projections. The venous wire is advanced from the inferior vena cava to the aorta, while applying short bursts of electrosurgical cutting. The wire is then ensnared, advanced proximally to the thoracic descending aorta, and exchanged for a rigid guidewire and the TAVR introducer. The valve is then advanced and deployed as per the protocol. The transcaval tract is planned for closure and the anticoagulation is reversed. A cardiac occluder device is placed sideways to abut the aortic wall. Finally, arteriography is performed to assess for persistent extravasation [57]. A detailed description of each step can be found in the state-of-the-art review by Lederman et al. and is beyond the scope of this review [57].

The transcaval access approach involves delivering an introducer sheath from the femoral vein and inferior vena cava across the retroperitoneal space to the infrarenal aorta. This technique utilizes the concept of permissive transcaval physiology where the aortic bleeding decompresses into the nearby inferior vena cava puncture site as both the retroperitoneal and aortic pressures are greater than the venous pressure. Therefore, it is crucial for there to be a venous opening for drainage at any point in time while there is arterial bleeding to avoid retroperitoneal hemorrhage [46,57].

#### 3.3.2. Outcomes

Transcaval access outcomes have been assessed through retrospective and prospective observational data. Two successive cohorts of 19 and 100 high-risk patients, who were ineligible for transfemoral, transapical, and transaortic accesses, underwent transcaval access and their outcomes were reported. Among the 19 patients of the first cohort, 17 had a successful TAVR, but 6 patients experienced vascular complications, 2 of whom required intervention. All patients had a persistent aorto-caval flow immediately after the procedure, and of the 16 who underwent repeat imaging within 1 week, 15 had complete closure of the aorto-caval tract [18]. In the second cohort, transcaval access was successful in 99 of 100 patients, and aortic closure with a nitinol cardiac occluder was successful in 98 of 99 patients. Inpatient survival was reported as 96%, and the 30-day survival was 92%. Major vascular complications possibly related to transcaval access were reported as 13%. The median length of stay was 4 days (range 2 to 6 days) and there were no vascular complications after discharge [58].

The transcaval approach has been compared to transaxillary and transfemoral access across eight centers in a propensity-adjusted analysis. This 4-year study described the outcomes of 238 and 106 patients who underwent transcaval and transaxillary approaches, respectively. The transcaval group had lower rates of stroke and similar bleeding when compared to transaxillary access. However, both approaches resulted in higher complication rates than transfemoral access. Limitations to this study included a lack of description on decision-making between transcaval and transaxillary access, increased utilization of the transcaval approach, and a higher stroke risk in the transaxillary group [59]. Overall, the limitations call into question the operator experience across all participating sites in the transaxillary access approach, and future comparisons of stroke risk are needed between centers with large volumes of transcaval and transaxillary access [60,61].

The current research suggests that transcaval access for TAVR is technically complex and requires mastery to be performed safely without a higher risk of retroperitoneal bleeding. This access type can likely be performed safely in high volume centers following mastery of the technique. However, this comes with a learning period and may potentially be best suited to centers looking to utilize this technique as the main alternative access method. We suggest that this alternative access may be considered for first-line alternative access only at centers that have adequate experience and a high volume with the transcaval approach.

#### 3.3.3. Patient Selection

Most patients can be considered for transcaval access as an alternative access site. Contraindications include the bowel positioned between the inferior vena cava and aorta, pedunculated aortic atheroma which may lead to embolization, and aortic dissection at the crossing target location. Interestingly, aortic ectasia and aneurysm are described as desirable zones to seat closure devices and are not contraindications [62]. For reasons discussed previously, this access should be utilized at high-volume centers with mastery of the technique to avoid complications. Prior to performing transcaval access, careful planning with contrasted CT of the abdomen and pelvis to identify a suitable target with a calcium free window on caval side of aorta, located away from important arterial branches, with no interposed viscera, and a suitable distance for the length of the introducer sheath, should be performed. A complete description of salient CT features is outside the scope of this review article and is extensively described by Lederman et al. [62]. A potential advantage to this approach over other alternative access sites is that it is primarily performed under conscious sedation.

### 3.4. Choosing the Right Access

The ideal alternative access for TAVR should be one that can be performed percutaneously, under conscious sedation, with local anesthesia, and with similar outcomes to the transfemoral route. Despite no current perfect alternative, there are multiple access sites which can be grouped into more invasive intrathoracic (transapical and transaortic) and less invasive extrathoracic approaches (transaxillary, transcarotid, and transcaval). Currently, there are no large randomized trials comparing outcomes in centers with mastery in alternative accesses, and therefore the recommendations are based on observational data. Table 1 compares the benefits, risks, pre-procedural planning considerations, and closure techniques used in intrathoracic and extrathoracic access.

In our opinion, centers may consider mastering at least one alternative approach and ensuring that the volume of cases affords maintenance of the technique prior to pursuing further expertise in additional approaches. Given the higher risk of complications and mortality with transapical and transaortic access, it may be reasonable to reserve these approaches for patients not suitable for extrathoracic accesses and prioritize expertise in transaxillary, transcarotid, or transcaval access.

**Figure 3:**
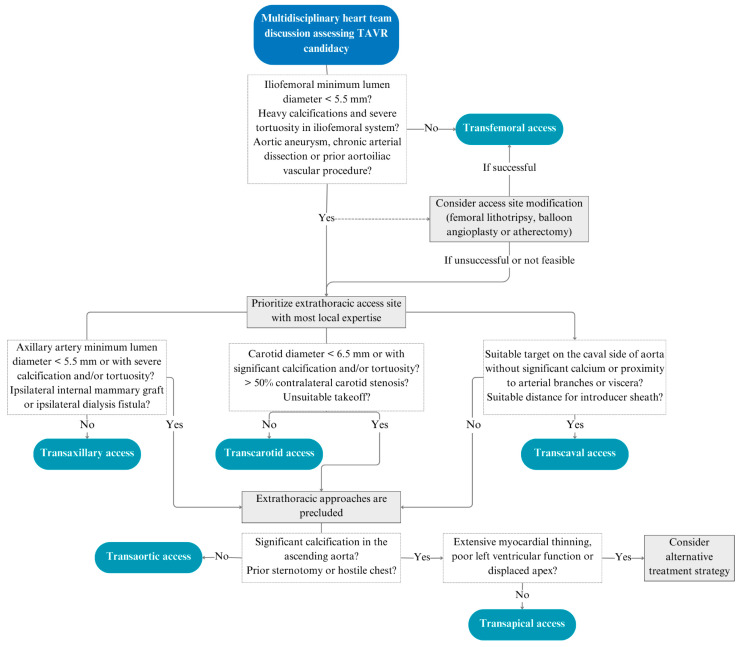
Alternative access algorithm. Proposed algorithm for choosing the approximate alternative access.

Based on this, we present an algorithm shown in Figure 3, where we recommend that patients are offered the extrathoracic access with most local expertise based on the patient characteristics. A transaortic access is considered only if the patient is not suitable for an extrathoracic approach, followed by a transapical access. If the patient is not suitable for these techniques, alternative treatment strategies are suggested. We also suggest considering referral to a center with mastery in a particular alternative access technique if this is deemed appropriate and more beneficial for a patient.

## Figures and Tables

**Table 1:** Comparison of benefits, risks, pre-procedural planning, and closure techniques of intrathoracic and extrathoracic alternative TAVR access sites.

Type of Access		Benefits	Risks	Pre-Procedural Planning	Closure Techniques
**Intrathoracic**	Transapical	Useful alternative when all other accesses are precluded	Considered the most invasive approach Requires thoracotomy and general anesthesia Unable to accommodate self-expanding valves Can be associated with damage to mitral valve apparatus and formation of left ventricular aneurysm	Imaging to rule out abnormalities in the regional thoracic wall or apical myocardium	Primary surgical closure
	Transaortic	Useful alternative when extrathoracic accesses are precluded Improved outcomes, when compared to transapical access (see text)	Requires partial sternotomy or mini thoracotomy Requires general anesthesia High risk of bleeding complications	Pre-TAVR imaging used to rule out: - Thorax deformities - Short ascending aorta (<6 cm) - Porcelain aorta - Patent coronary venous bypass graft anastomosed to ascending aorta	Primary surgical closure
**Extrathoracic**	Transaxillary/ transsubclavian	Conscious sedation can be used (with percutaneous approach) Improved outcomes, when compared to intrathoracic accesses (see text)	General anesthesia is required (with surgical cutdown approach) Axillary/subclavian arteries are more prone to dissection (see text)	Pre-TAVR imaging used to confirm: - Axillary artery diameter > 6.5 mm - No significant calcification or tortuosity - Absence of patent internal mammary graft	Perclose sutures (with percutaneous approach) Primary surgical closure (with surgical cutdown approach)
	Transcarotid	Better device control and positioning (direct and short distance to aortic valve)	General anesthesia is required (majority of cases done with surgical cutdown approach)	Pre-TAVR imaging must include carotid and vertebral ultrasound and CT/MRI of brain, used to: - Assess carotid artery size, tortuosity and calcifications - Rule out contralateral carotid stenosis of >50% - Rule out ipsilateral vertebral artery occlusion or prior carotid intervention - Confirm patency of the circle of Willis and collateral cerebral blood flow	Primary surgical closure of carotid artery
	Transcaval	Conscious sedation is typically used	Steep learning curve Risk of retroperitoneal bleed Risk of creation of AV fistula Requires occluder device to be placed in aortic wall	Pre-TAVR imaging used to ensure: - Calcium free window on caval side of aorta - No interposed abdominal organs - Target window away from aortic branch vessels	Occluder device placed in the aortic wall Venous hemostasis is achieved
